# “That Came Back to Haunt Me”: Violence Against Women Survivors’ Concerns About Police Use of Body-Worn Cameras

**DOI:** 10.1177/08862605241311610

**Published:** 2025-01-07

**Authors:** Amanda Couture-Carron, Alana Saulnier

**Affiliations:** 1 Rowan University, Glassboro, NJ, USA; 2 Queen’s University, Kingston, ON, Canada

**Keywords:** violence against women, sexual assault, intimate partner violence, body-worn cameras, police

## Abstract

Despite the substantial contact police have with survivors of violence against women, empirical accounts of survivors’ perceptions of police use of body-worn cameras (BWCs) are limited. This study examines survivors’ concerns with BWCs. We present qualitative data from semi-structured interviews with 33 survivors of intimate partner abuse and sexual assault. While the majority (79%, *n* = 26) of the women in this study support police use of BWCs, most (90%, *n* = 30) still express concerns with the technology. Survivors’ concerns fell into three main areas: fear of BWCs capturing trauma responses that could be used against survivors, BWCs decreasing survivor comfort and reporting, and BWCs revictimizing survivors and contributing to survivors’ loss of control. The findings reveal concerns that police can work to address to avoid survivor–police relations being deteriorated by police use of BWCs.

## Introduction

Police adoption of body-worn cameras (BWCs) is widespread across western nations; at least 20% of Canada’s police services (Saulnier & Abbatangelo, 2024), half of law enforcement agencies in the United States of America (USA) (Hyland, 2018), and all police forces in the United Kingdom are estimated to use BWCs (Lum et al., 2020). Advocates contend that BWCs will curb—or, at least, document—police misconduct (Ariel et al., 2016; Chavis, 2016; Coudert et al., 2015). BWCs have the potential to increase police transparency and accountability, and, therefore, public trust (Alpert & McLean, 2018; Bud, 2016). BWCs are also valuable to police as a tool that supports training (Lum et al., 2019) and that can deter or refute false complaints (Davies & Krame, 2023). These expected benefits are founded on the ability of BWCs to document officer and public interactions. However, the implications of BWCs for violence against women (VAW) survivors have received little attention given the substantial contact police have with this group. Studies find that 30% of all reported violent crimes in Canada are cases of intimate partner violence (IPV) (Conroy et al., 2021), and another 10% are sexual assault cases (Department of Justice Canada, 2021). While both men and women and frequently the victims of these crimes, meta-analyses demonstrate women experience these offenses at particularly high rates (Dworkin et al., 2021; Spencer et al., 2022). Research out of Australia found that BWCs were most often activated during family violence calls for service (Iliadis et al., 2021). Taken together, VAW survivors interacting with police are likely to increasingly be recorded by BWCs. Police play a significant role in responding to VAW. They are the criminal justice system’s (CJS) frontline response to VAW, and a primary means of protecting survivors (Leisenring, 2012). As such, it is important to understand survivors’ concerns with police use of BWCs, and how BWCs may impact their experiences with police, including their willingness to report violence.

Concerns about police use of BWCs during interactions with VAW survivors have been broached (e.g., Adams & Mastracci, 2017; Harris, 2020; Iliadis et al., 2023, 2024a, 2024b), but additional empirical research is needed to validate these concerns. These concerns are highly pertinent to consider within Canada, where a growing number of police services are adopting BWCs,^1^ but are relevant everywhere VAW is found. Literature weighing in on the impact BWC use may have on survivors has largely spoken *for* survivors without speaking *to* survivors (for exceptions see Barlow, 2023; Iliadis et al., 2024a; Saulnier et al., 2022). To continue addressing this knowledge gap, the current study examines survivors’ concerns with BWCs. We present qualitative data from semi-structured interviews with 33 survivors of VAW documenting their concerns with police use of BWCs. The women in this study were survivors of intimate partner abuse and/or sexual assault. Most had interactions with police regarding their VAW experiences and a few had experiences with officers wearing BWCs. Although the majority of participants support police use of BWCs (*n* = 26),^2^ almost all (*n* = 30) still express concerns with the technology. Our objective is to bring awareness to survivors’ concerns so police can identify mitigation strategies that avoid BWCs undermining police-survivor relations.

## Literature Review

### Survivors’ Interactions with Police

Survivor–police relations are often tenuous. Recent estimates suggest that only 20% of survivors of spousal violence^3^ (Conroy, 2021) and 5% of survivors of sexual assault (Department of Justice Canada, 2021) report to police. Survivors hesitate to report their victimization to the police for numerous reasons, but common concerns relate to the police response itself. For example, survivors express concerns about: police minimizing their abuse; police blaming, shaming, and judging them; and, whether police can or will do anything about the victimization (Carbone-Lopez et al., 2016; Decker et al., 2019; Johnson, 2015, 2017). For survivors who have reported to police, their experiences vary. Some women are satisfied with the police response and some are not, citing experiences with unsympathetic and unhelpful officers (see Johnson, 2017; Saxton et al., 2018; Sphon et al., 2015; Vopni, 2006). Negative experiences with police during reporting are a factor that fosters revictimization (Campbell et al., 2001). Revictimization is the “trauma, distress, and alienation” or “second assault” survivors may experience after an assault that stems from poor treatment by the CJS or other service providers (Maier, 2012, p. 289). Given the existing low levels of VAW reporting and the harm that can follow negative interactions with police, it is important to understand the potential effect BWCs may have on police-survivor relations.

### BWCs and Violence Against Women

A central rationale underlying police adoption of BWCs was that recording police-public interactions would have a “civilizing effect” on those encounters (White, 2014). Public perceptions research demonstrates that public support for police use of BWCs is based, in part, on expectations that BWCs will improve officer behavior and professionalism (Crow et al., 2017; Northeastern University, 2018; Toronto Police Service (TPS), 2016; White et al., 2017), the quality of police-community interactions (Kopp & Gardner, 2021), and police accountability (Taylor et al., 2017; TPS, 2016). Experimental field studies further demonstrate that people tend to evaluate police interactions that involve a BWC more favorably than those that do not (Demir, 2019; McClure et al., 2017; Saulnier et al., 2020). The empirical research exploring crime victims’ attitudes toward BWCs mirrors these findings, again demonstrating high levels of support for police use of BWCs that are related to feelings of comfort and safety (Goodall, 2007; TPS, 2016). The few studies that have focused on VAW survivors similarly reveal that survivors tend to support police use of BWCs underscoring the extent to which BWCs promote feelings of police accountability (Iliadis et al., 2024a; Saulnier et al., 2020). In addition, some have speculated that having video footage of a survivor’s statement might reduce demands on the survivor to have to retell their story either to multiple officers or in court, thereby reducing opportunities for revictimization (Harris, 2018; Iliadis et al., 2024a; Yeong & Poynton, 2017). However, other scholars have noted that such expectations may leave survivors disappointed. For instance, in Canada, while BWCs document the process of making a report to a frontline officer they do not substitute for subsequent interviews with investigators or prosecutors (Saulnier et al., 2022).

BWC footage has also been characterized as a “game changer” for courts (Needham, 2015). BWC footage brings arbitrators of justice more information by visually documenting details of the scene including injuries and the demeanors of those involved, which can increase the likelihood of guilty pleas and convictions (Drover & Ariel, 2015; Harris, 2018). Surveys polling police (Gaub et al., 2016; TPS, 2016) as well as prosecutors (Merola et al., 2016; Westera & Powell, 2017) demonstrate both groups are optimistic that BWC footage will help produce convictions, particularly for IPV cases. In one study, prosecutors report that visually capturing survivors’ initial reports when emotions are high is a compelling form of evidence that can support survivors’ credibility (Westera & Powell, 2015). Survivors have also reported that BWC footage contributes to their credibility by providing “objective” evidence that supports their account of the abuse (Iliadis et al., 2023, 2024a, p. 18). Moving beyond these assumptions, empirical evidence supports the value BWC footage brings to prosecutions. Assessments of the effects of BWCs on court outcomes are limited but studies from countries including the United Kingdom (U.K.), U.S., and Canada have found BWCs support prosecutions (Ellis et al., 2015; Goodall, 2007; Morrow et al., 2016; Saulnier et al., 2020), though other evaluations have found that video footage does not increase convictions (Yeong & Poynton, 2017). However, of particular note is a collection of studies finding that VAW cases with BWC footage are more likely to lead to arrests, charges filed, court processing, and result in guilty pleas and guilty verdicts (Grossmith et al., 2015; Katz et al., 2014; Morrow et al., 2016). By introducing a new form of evidence, BWC footage is expected to support prosecutions by limiting perpetrators’ ability to pressure survivors to recant (Harris, 2018; Yeong & Poynton, 2017) as well as standing in for survivors if the survivor withdraws from the prosecution process (Grossmith et al., 2015; Morrow et al., 2016). However, while BWCs present a solution for holding offenders accountable in the absence of victim cooperation, leveraging BWC footage to force an outcome that a survivor does not want can create tensions between the survivor and the CJS (Ellison, 2018; Fan, 2016), and raises questions about whose interests BWC serve (Barlow, 2023).

There are other unintended consequences of police use of BWCs for survivors. For example, BWCs may exacerbate fears related to shame by visually capturing a survivor’s report. BWCs may record survivors when they are in a state of shock, intoxication, undress, or in other potentially embarrassing conditions (Murphy, 2015). Research indicates that some survivors think BWCs can contribute to their humiliation (Iliadis et al., 2024a). Murphy (2015) suggests that survivors may feel ashamed of family and friends seeing the footage. As such, BWCs have the potential to exacerbate survivors’ distress and trauma, a possibility supported by Ilaidis et al. (2024a) work, which reported that some survivors did feel BWCs intensified their trauma and made them feel upset. In addition to capturing survivors in vulnerable states, survivors may also be concerned about BWCs scrutinizing their private spaces. “BWCs bring state surveillance” into survivors’ lives during a traumatic and deeply personal moment (Adams & Mastracci, 2017, p. 324). By capturing survivors’ trauma and their private spaces, BWCs are arguably more intrusive than traditional police approaches, such as taking written statements at the scene or video statements at a police station. Indeed, some survivors have described BWCs as intrusive and degrading (Iliadis et al., 2024a). The presence of cameras in survivors’ private spaces—often their homes—may cause women to hesitate to call the police, fearing that BWCs may capture evidence of illegal or socially undesirable behavior (Adams & Mastracci, 2017).

In addition to the intrusion, BWC footage may not “play well in court” for a variety of reasons related to the scrutinization of survivors (Adams & Mastracci, 2017, p. 318). First, assaulted women’s responses to trauma may contradict expectations of how a “true” victim should behave, undermining their credibility (Randall & Haskell, 2013). Survivors may be scared and appear angry or irrational when initially reporting—the opposite of the “ideal” victim (Christie, 1986). If captured by a BWC, this may show a survivor in an unfavorable way, negatively impacting CJS professionals’ perceptions of them (Douglas & Goodmark, 2015; Harris, 2020; Iliadis et al., 2023, 2024a). Although some prosecutors believe documenting heightened emotions builds survivors’ credibility (Westera & Powell, 2015), capturing initial emotional responses may undermine their credibility (Iliadis et al., 2023). Second, BWC footage may also capture inconsistencies or survivor’s attempts to minimize their abuse; common responses to trauma and VAW (Harris, 2020; Randall & Haskell, 2013). In court, these inconsistencies, if captured by video, would fuel defense attorneys’ already combative treatment of survivors (Johnson, 2017), including threats of perjury charges (Harris, 2020). Third, abusers have been known to alter their behavior to manipulate the police into believing they are the victim rather than the perpetrator (Douglas, 2019; Harris, 2020; Mandel et al., 2021). This concern is echoed by survivors who have reported abusers appearing calm in BWC footage (Iliadis et al., 2024a, 2024b). Iliadis et al. (2024b) note that footage showing abusers behaving in unexpected ways, particularly when positioned in relation to a survivor that is not presenting as “ideal,” may hamper prosecution efforts. As Adams and Mastracci (2017) argue, BWCs do not distinguish survivors from abusers. Finally, evidence suggests that the presentation of visual extra-legal information (e.g., contents of a home) can have a prejudicial impact on jurors’ perceptions in IPV cases, including reducing the odds of a defendant being found guilty (Smith et al., 2019).

As the primary response to VAW and the “gatekeepers” to the CJS and other supports, the effects of police use of BWCs on police-survivor relations are critical to understand. The implications of police use of BWCs for VAW survivors are expected to be complex and nuanced. Survivors of VAW are a key source of information on these effects.

## Methods

This study uses qualitative data from 33 semi-structured telephone interviews with survivors of VAW, specifically women who experienced sexual assault and/or abuse by an intimate partner. Participants were recruited with the help of victim services and victim advocacy groups across Ontario, Canada that shared an advertisement for the study (e.g., through social media, listservs, and bulletin boards) ^4^. Interested participants contacted the principal investigator to arrange a telephone interview. Telephone interviews provide participants “pseudo-anonymity,” which is thought to increase comfort when discussing sensitive topics (Glogowska et al., 2011, p. 22), and allow for the recruitment of a larger sample.

To qualify for participation, participants had to self-identify as women, over the age of 18, and survivors of VAW. Contact with a police officer was not necessary for participation as the objective of this study was to explore women’s concerns with contacting police. Thus, survivors who had not contacted the police have valuable feedback to offer. Most participants, however, did have contact with the police related to their victimization. Further, our interest is in survivors’ opinions on police use of BWCs rather than their experiences with officers wearing BWCs. While restricting the sample to persons who had interacted with an officer using a BWC would have offered valuable insight through lived experience, doing so would have severely constrained the number of prospective participants given that BWC use in Ontario was limited to a few pilot projects at the time of this study. We acknowledge that an interaction with an officer wearing a BWC may shape survivors’ perspectives of police use of BWCs, but we also maintain that survivors’ general opinions and concerns still offer valuable insights.

The authors conducted the interviews, which were audio recorded with participants’ consent. Prior to and throughout the interview, the interviewers reminded participants of the voluntariness of the study. The interviewers also assured participants that their decision to participate would remain confidential and would not affect their relationships with any victim service groups or police services. A letter of intent describing the study and a follow-up thank you letter were provided to all participants. These letters provided contact information for support services if the participant experienced distress.

The interviews were approximately 30 to 90 min and included closed- and open-ended questions about the participants’ demographics and background, their perceptions of and experiences with police, and their opinion of police use of BWCs. For example, the questions sought information on whether police use of BWCs would impact their decision to report victimization, how they would feel about their interactions with police being recorded, and what advantages and disadvantages police use of BWCs posed for survivors. The semi-structured format of the interview allowed interviewers to encourage participants to expand on their responses and discuss related issues as they arose. Participants received a $40 (CAD) gift card as a token of appreciation for their time.

The audio-recorded interviews were transcribed verbatim. The authors then thematically analyzed the data in NVivo using a multistage process of open, axial, and selective coding. We used an inductive approach whereby the themes emerged from the data rather than existing research (Braun & Clarke, 2022). Each researcher brought familiarity with the data to the analysis process from their experiences conducting the interviews. The authors co-developed an initial set of codes and then engaged in a line-by-line analysis of the transcripts together. During this process, the authors revised the coding scheme as necessary and further developed the scheme with subthemes.

## Results and Discussion

### Participant Profile

Thirty-three women participated in this study. Participants ranged in age from 19 to 59 years with the average age being 37 years. All participants, except for three, were born in Canada. The majority (70%, *n* = 23) identified as White. The remainder (30%, *n* = 10) identified with other racial/ethnic groups, including four as Black and three as Metis.

The sample was highly educated. The majority (55%, *n* = 18) had at least some college education while 18% (*n* = 6) had a university degree and 15% (*n* = 5) had a master’s degree. Roughly 12% (*n* = 4) had a high school diploma or less. At the time of the interview, three-quarters of the women had a household income of less than $50,000. Most (48%, *n* = 16) reported working as their main activity for the last 12 months and the majority (73%, *n* = 24) indicated their main source of income was from working.

### Survivors’ Perceptions of BWCs

Prior to discussing the concerns survivors have with BWCs, it is worth recognizing that the majority (79%, *n* = 26) of the women support police use of BWCs. Only one participant opposed BWCs and six were unsure whether they supported police using BWCs. Although beyond the scope of this paper, reasons for this support revolve around increasing police accountability, generating evidence, and increasing convenience (see Saulnier et al., 2020 for these results). While support for BWCs is high, it is caveated with concerns. Thirty of the participants described concerns associated with police use of BWCs that fell into three main themes: (a) BWCs capturing trauma responses, including statement inconsistencies, that could be used against survivors; (b) BWCs decreasing survivors’ comfort and reporting; and (c) BWCs revictimizing survivors and contributing to survivors’ loss of control.

#### BWC Footage Being Used Against Survivors

Survivors’ behavioral responses to trauma may clash with stereotypical perceptions of “true” victim behavior (Randall & Haskell, 2013). Survivors may appear irrational, make inconsistent statements, or attempt to minimize or deny the abuse they experienced (Douglas & Goodmark, 2015; Harris, 2020; Randall & Haskell, 2013). Having personally grappled with such issues, many survivors in this study (33%, *n* = 11) expressed concern about how their trauma would be interpreted if captured on BWC footage. These women worry that survivors may be seen as acting in “abnormal” or unexpected ways. For example, Cheryl^5^ explained,

When something traumatic happens to you, you are not in the best state of mind. Right? You’re experiencing the effects of trauma in the moment and you go into fight, flight, or freeze. . .the trauma responses and what happens to people in the moment. . .and sometimes you don’t always say everything because you’re in such shock. . .you can have various reactions that may seem outside the realm of what would be “normal” for somebody in a situation like that.

Ava explained that trauma affects how survivors may appear on video,

You’re also losing your mind while that’s happening. . .At that time, your brain is firing differently, right. Like even me watching it, my sentences, like I wouldn’t finish a complete sentence and then I’m on to something else that really didn’t fit at that time. . . PTSD [post traumatic stress disorder] and a traumatized brain and shock, and fricking pain. . .I wasn’t drunk but you can look drunk if your train of thought isn’t there. . .

When survivors present in non-stereotypical ways, they are seen as less credible (Ask, 2010; Franklin et al., 2020). While these trauma responses would have only been witnessed “live” by responding officers prior to the implementation of BWCs, recording the encounter means these responses are accessible to a wider audience, such as defense attorneys, judges, and juries. In Canada, most police services that use BWCs require officers to active their cameras as soon as reasonably possible during all investigative contacts with the public^6^ (Saulnier & Abbatangelo, 2024), meaning that the entirety of a contact with a survivor would be recorded. Canadian police are legally required to disclose all information gathered related to an offense that is being prosecuted to the Crown Attorney (prosecutor) (Brucker, 1992) who is, in turn, required to disclose all information pertinent to the case to defense counsel, who may choose to use any of that content in their own case (R v Stinchcombe, 1991). Survivors in this study were aware of the potential for trauma responses to undermine survivor credibility and recognize the heightened risk that capturing those reactions with BWCs may pose, consistent with findings from Iliadis et al. (2023, 2024a). For example, Tina indicated that observers can misunderstand trauma responses,

[Victims] could appear less credible, they might not be, you know, the. . .perfect victim. . .and then that kind of colors the way they’re treated through the rest of the criminal justice system. . .what you’re seeing is a completely broken down, traumatized victim. And that can be misunderstood unless people really get what happens, like unless they’re really. . .well like trauma informed, right? They really understand how this experience can affect a person at that time.

BWCs document initial police contact in a more visceral way. This includes BWCs capturing details that might have been entirely absent from the record if not for the technology’s use. For instance, participants drew attention to the consequences of BWCs capturing unconventional body language on survivors’ credibility. As Vera worried,

Like am I fidgeting a lot? That makes me look like I’m lying about something, or, you know, just body languages experts, would they see certain things that maybe they would read into too much, or something like that. . .I don’t know how I would be perceived by other people looking at this video. They could either take it either way. They could be like, “oh, she’s putting on a complete show” or “this girl’s actually legit, and she’s going through pain.” You know what I mean? So, it all depends on how the people perceive it.

Aubrey similarly worried that footage of body language and non-stereotypical responses may make observers skeptical,

There is always the potential of people watching that and coming to the conclusion that, like they bring to light certain things that you’re doing and saying like, “Oh, she was frantic. Look at how frantic she was.” Or “Oh, she’s darting her eyes back and forth so maybe she’s not telling the truth.” Or “She’s not crying enough, so maybe that’s not right.” People just assume if you’re telling a traumatic story, they’re going to see it and you’re going to be crying and like super upset about it but if you are not that type of person and your way of telling a traumatic story is to shut yourself off from it, people can take that as like, oh maybe it’s not true then because it’s not looking like it’’s true. So, there’s always that chance of it causing skepticism when you aren’t responding the way that they would assume you should.

In addition to general concerns about the presentation of trauma and its potential to undermine credibility, participants also worried that defense attorneys could use footage of survivors’’ behavior (36%, *n* = 12) and inconsistent statements (12%, *n* = 4) against them in court. This fear is justified given that defense attorneys regularly argue that signs of trauma and statement inconsistencies are indicators of deception (Ellison, 2005). Multiple survivors speculate that defense attorneys would present manipulative interpretations of trauma captured on BWC footage to undermine their credibility. For example, Jennifer—who was recorded by a BWC—discussed how this happened to her,

I was made to look like the liar in that situation from the body-worn camera because they said what the footage had on it made me look delusional. . .they would’ve had me freaking out on camera and go like, “yeah, she’s crazy, don’t listen to her, send you to the psych ward.” And then they think you’re crazy because they have you on video freaking out, about telling them a “story” instead of the situation; when the situation is actually not a story.

Jennifer’s statement demonstrates how a defense attorney could present footage of trauma responses in a way that suggested she should not be believed. Even if she was composed during her court testimony, footage of her immediately following a traumatic event has the potential to cast doubt on the veracity of her statements. Linda also indicates this could have happened in her situation. She explained that she heatedly expressed frustration during a negative experience with police, underscoring that if the interaction had been recorded, her abuser or his attorney could have used the contents against her,

I think that’s the disadvantage because that’s all based on emotions as a way they’re acting, right. . .because I’ve walked out of a police station saying, “Well, fuck this then.” You know what I mean? “Ok, fine. Fuck you.” I’ve actually said that. If they had body cams at that point, it may make me look bad in court. . .the accused will take that video and say, “Look, she’s crazy. Look at how she’s responded to the police.”

The participants’ concerns for how defense attorneys could manipulate BWC footage to undermine the credibility of the survivors and potentially paint them as the aggressors aligns with concerns raised by Iliadis et al. (2024a, 2024b).

Alongside counterintuitive emotional responses to trauma, inconsistencies in descriptions of events are also common when reporting sexual assault and abuse (Johnson, 2017; Randall & Haskell, 2013). Like non-stereotypical trauma responses, defense attorneys use even minor inconsistencies, omissions, and errors in women’s statements to undermine their credibility (Ellison, 2005; Quilter et al., 2023; Tosto & Bonnes, 2022). BWC footage more readily documents such occurrences and may be used to provide support for defense attorneys’ claims that a survivor is inconsistent and, thereby, not credible. Consistent with Adams and Mastracci’s (2017) speculation and Iliadis et al. (2023) findings that BWCs may disadvantage survivors by capturing inconsistencies typical in initial reporting, multiple women in this study worried defense attorneys would use BWC footage documenting inconsistencies to discredit them. For example, Natalie reported,

Well, sometimes when you’re first reporting and you’re in the heat of what has happened, it’s hard to get the facts straight. When you’re in trial, it’s important to have your facts straight and you want to appear that you are in control and that you know exactly what happened, and so, I would want to be careful about that because as a victim, it’s hard to make sure that you’ve got it correct. You need the processing time.

In addition to unintentional inconsistencies, it is not uncommon for reports of VAW to contain *intentional inconsistencies* as survivors of abuse sometimes change their stories or minimize their abuse to protect their abusers (Cervantes & Sherman, 2021; Dunham & Senn, 2000). Such inconsistencies can occur out of fear of their abuser or as an effort to protect their abuser or their relationship. Multiple women in the study feared abusers’ lawyers could use intentional misrepresentations of abuse to discredit survivors. Ava described how her abuser’s lawyer used her inconsistent statements recorded by a BWC against her,

I certainly got nailed in court. . .his defense team put those two cameras up that I said, “Officers, no. I fell, I wasn’t abused.” You know that sort of thing, like that came back to haunt me. . .seven hours of footage gives a whole lot of options for how to destroy a victim on the stand. If there was an inconsistent statement at any point. . . I was on the stand for three days as a victim, and they rip you apart. So, there’s a risk to cameras on at all times. . .like it was just more of the same to make me look like a liar. So yeah, it certainly gives the defense some fuel, right? So, there is a risk, I’m sure. That’s in my opinion, but you are looking straight into a police officer’s eyes and saying, “This didn’t happen.” That’s powerful to a jury.

Natalie expressed these fears as well,

So, when a victim is initially, and certainly for my case, when you’re initially with your spouse and you’re trying to protect that relationship, and. . .you’re not ready to leave that relationship, the victim can then sometimes make statements to protect that relationship. And so, if those statements were then later on used against them, that would be very damaging to their whole case. So, they would appear as though they’re not reliable.

Similarly, Tracey explained,

People could say things and then change their story later on. . .because they want to protect somebody. . . when you change your story then it’s going to make it more suspicious.

Taken together, the statements from the women in this study indicate that BWC footage capturing survivors’ trauma may, unintentionally, strengthen defense attorneys’ existing efforts to discredit survivors. This concern is further highlighted by Michelle’s statement,

They want to use everything against you to poke holes in your story. . .like maybe you were arguing with the officer. . .[it] could cast you in a shadow, in a negative light. . .you might be freaking out. You might do some incriminating things. And I think that might reflect negatively in a system that already seems to reflect negatively on victims in court. . .things could be taken out of context; things could be used against a victim.

#### BWCs Undermining Survivor Comfort and Reporting

Discrepancies between official statistics and self-report studies suggest that abused women often do not report abuse to the police (Conroy, 2021). Feelings of embarrassment and shame are some reasons why women do not report (Carbone-Lopez et al., 2016; Davies et al., 2007; Liang et al., 2005). Women in this study worry that BWCs may exacerbate these feelings and further discourage reporting. Specifically, participants expressed concerns that BWCs may make survivors uncomfortable (48%, *n* = 16), which may cause them to limit or censor what they tell the police (33% *n* = 11). For example, Brittany said, “I would probably feel uncomfortable, mostly embarrassed, because it’s embarrassing.” Similarly, Angela explained,

I would be very uncomfortable [with a BWC recording] because it was a very traumatic situation and just kind of knowing how our bodies and brains react to trauma. . .knowing myself, when I’m just stressed out or nervous or anxious or any kind of negative emotion, I don’t want to be on camera.

Jennifer, who was recorded by a BWC, described how the BWC made her feel deeply uncomfortable and put on the spot. Her comments demonstrate how police use of BWCs can make an already difficult situation more distressing,

I can honestly say it was the most uncomfortable. . . I felt like I was being put on the spot. . .I didn’t want to look at them. I don’t think I looked at them straight in the camera or straight in the face when they were interviewing me. I kept my head down or something like that. I don’t like my face being shown when I’m telling my personal stories like that, because I feel like my face is being exposed. . .It made me feel very uncomfortable and put on the spot and it made me feel like. . . almost like a kid in a talent show with the spotlights beaming on them, about to sing a song, but then they just freeze. Like a deer in headlights, that’s all it feels like when I feel like I’m on camera.

Multiple women spoke about BWCs leaving survivors feeling “exposed” or “embarrassed.” Not only do cameras capture survivors recounting sensitive events that they may wish to keep very private, but they also contribute to survivors’ feelings of vulnerability simply through the act of recording. This perspective aligns with Iliadis et al. (2024a) findings that some survivors feel BWCs are intrusive and degrading, contributing to their feelings of humiliation.

Aubrey explains how BWCs may cause further discomfort to already vulnerable survivors, especially survivors who may have been under surveillance by their abuser. She said,

I think, as a victim, if you are talking to a police officer after calling them, after dealing with any type of abuse, that can be incredibly off putting to know that what you’re saying is being recorded when you’re in an incredibly vulnerable position. . .there’s a lot of victims out there. . .that have this really uncomfortable feeling about being recorded because they feel like everybody’s always watching what they’re doing. . .I think it might have put me a little bit more on edge.

Moreover, BWCs may undermine women’s already tenuous feelings of safety when seeking help. As Sharon explains, “[A BWC] doesn’t make you feel safe as a victim and comfortable to even speak to [police].” While BWCs may help survivors feel more comfortable as some studies have found (Goodall, 2007; TPS, 2016) they may also actively intimidate survivors. For example, Michelle said,

I wanted our meetings to be a safe space where I could ask [the officer] questions and not think, you know, “Oh my God, is this going to be reported?” But in my specific experience, [a BWC] would have intimidated me because I was unsure, I was so unsure about the situation.

The discomfort BWCs may cause was connected to concerns that BWCs may diminish survivors’ willingness to disclose or prompt self-censoring. For example, Grace explained,

If they were here questioning [with BWCs] I probably wouldn’t have said too much. . .because, I know this sounds crazy, but I’m just not comfortable. . .I feel like I would be more self-conscious about every word that came out of my mouth. . .I just would’ve probably left. . .I wouldn’t speak as much.

Similarly, Sharon said, “In some instances, people might watch what they say.” BWCs may cause survivors to be more selective about what they tell police out of concern for how they may appear. As Cheryl speculates, “I feel like I might censor myself. . .because you do worry about how you’re perceived. I do anyway. So, I think I would be embarrassed.” Likewise, Heather indicates that she would not have been forthcoming with the police had they been wearing a camera,

Being able to open up to talk to [police] about the situation. . . I know if they had a camera at the time, I definitely wouldn’t have. I would’ve felt a little bit more scared than I was, and I would’ve been just afraid.

Many women in this study believe BWCs will make survivors feel more uncomfortable when reporting abuse—uncomfortable enough that survivors may limit disclosing details of their abuse. Survivors often feel ashamed and embarrassed when reporting abuse (DiMauro & Renshaw, 2021; Griffin et al., 2022; Johnson, 2015, 2017). Participants suggest capturing survivors’ reports on video may exacerbate those feelings. The potential for BWCs to undermine women’s willingness to report victimization is troubling given that VAW is already an underreported crime. Canadian national victimization surveys routinely find that VAW is underreported; recent studies report that 64% of victims who are women do not report IPV (Burczycka, 2016) and 83% of victims who are women do not report sexual assault to the police (Department of Justice Canada, 2019).

#### BWCs, Revictimization, and Loss of Control

Beyond causing survivors general discomfort, women in this study express concern that police use of BWCs may revictimize survivors (42%, *n* = 14) and contribute to a loss of control (24%, *n* = 8). Survivors’ loss of control and revictimization go hand-in-hand. The loss of control contributes to revictimization experiences (Maier, 2008). The use of BWC footage to attack survivors’ credibility, as previously discussed, is one way that BWCs can contribute to survivors’ revictimization. Many women in this study, however, are also concerned that simply being recorded and having to watch the footage may revictimize survivors. For instance, Zoe explains,

I just think that a lot of assault victims feel that they’re almost being assaulted again. . .I think that you can feel violated maybe to have that camera on.

Stella also expresses this concern, saying, “I mean, being recorded like that would be. . . I would feel violated.”

For other participants, the prospect of revictimization stems from having to watch the footage at a later point. According to these participants, watching the recordings would bring the trauma back to the fore. For instance, Brittany said,

I would probably feel revictimized just because. . .I feel [BWC footage] would kind of just like bring back all the information and I would just kind of, just revictimize myself all over again.

Heather made a similar statement, saying, “having to re-watch that is a little bit traumatizing and brings back the victimization.”

Many participants characterized the use of BWCs as yet another challenge to deal with during an already difficult situation. For example, Cheryl worried that being recorded would exacerbate what she was going through,

Am I going to be subject to more degradation. . .or vulnerability or embarrassment or, you know, feeling more worthless? Because that’s what happens as a victim.

This sentiment is also illustrated well by Angela’s statement,

Having already, like, been going through a traumatic experience and then going in to talk to police. . .was kind of like the negative experiences I’ve already had. . .my sexual assault. . . So, it’s like, I feel like it would just be really difficult on me and I feel like it would. . . like even recording would be another traumatic experience for me.

While BWC use is intended to be helpful, it is important to recognize a BWC may introduce distress for a survivor, and that distress may be exacerbated by BWC policy choices that deny survivors’ control (e.g., denying survivors consent options; Saulnier et al., 2022). Many Canadian police services with BWCs require officers to record whenever interacting with a member of the public (notifying parties if possible; Saulnier & Abbatangelo, 2024). In this situation, the officer may represent another person who does what they want without care for the survivor, which contributes to a loss of control, as described by Sharon,

Most times when you’re a victim, things are being done to you without your own good will. When you are going to the police you’re looking for, like, a safety net. . .there’s just someone else that doesn’t really care and. . .they want to record you so they’re going to record you. It’s like another upset, like it doesn’t make you feel safe as a victim and comfortable to even speak to them.

This loss of control extends beyond the situation to include loss of control over the footage itself. Participants speculated on who would have access to the footage, where it would be stored, to whom it would be shown, and how it would be used. In general, participants expressed fears about what would be done with the footage. As Brittany simply states, “I feel you’re not in control of this information.” Cheryl offers a more detailed explanation,

I would be worried that, you know, where is this going to be used? Who’s going to view it? Could [the abuser] see it? I guess there would be some worries and concerns with that, and just sort of like wondering where it would end up. I know generally laws of confidentiality and how that probably wouldn’t happen, but I think that in that mind state and in that sense of chaos, it could be really scary. . .just the risk to the vulnerable person, the person who experienced the traumatic violent incident. Just adding, you know, where is that video going to be seen?

The legal obligations of police regarding data storage are not always clear to the persons they are interacting with. The legislative landscape on such matters can be highly fragmented depending on the country of focus. For example, in Canada, public agencies such as police services are subject to privacy legislation based on the level of government at which they operate (municipal, provincial, or federal). For instance, police services operating at the municipal level in the province of Ontario are subject to the *Municipal Freedom of Information and Protection of Privacy Act* (MFIPPA) (Ontario, 1990). Guidelines like MFIPPA provide high level regulation (i.e., data meeting the definition of personal information is highly protected from public disclosure; data meeting particular thresholds must be stored for a minimum period), but the specifics of where, how, and how long BWC data are stored are largely determined at the service level. As the above quote demonstrates, a lack of knowledge on these issues exacerbates an existing lack of control many survivors experience because of their victimization. This was echoed by Grace,

We already have lost control as victims, and I’d hate to have more loss of control. . .the gravity of it all was just already. . .it’s already huge, it’s already overwhelming, and to have at least a little control is helpful.

The potential for police use of BWCs to revictimize survivors needs to be weighed against the decision to use BWCs. Recording a survivor without their consent could be experienced as a “second assault”. The lack of choice and prioritization of the needs of the police over the survivor can be interpreted as evidence of the “unresponsive” treatment normalized by the CJS (Campbell & Raja, 1999). Responsive treatment prioritizes meeting survivors’ needs with empathetic care (Martin & Powell, 1994), established by providing survivors with control, empathy, respect, and safety (Murphy-Oikonen et al., 2022; Saulnier et al., 2022; ten Boom & Kuijpers, 2012). Failing to meet these needs can be psychologically harmful to survivors, resulting in feelings of guilt, depression, anxiety, and hesitancy to seek further help (Campbell & Raja, 1999, 2005).

## Conclusion

Fueled by demands for police reform and accountability, the implementation of BWCs is one of the most significant changes to policing in the last century (White & Malm, 2020). In both public debates and academic research, there is an assumption that any potential advantages or disadvantages of BWCs will be the same for all who interact with police. However, survivors of VAW are one group that BWCs may differentially impact. Attention to survivors is important because many police-reported violent crimes involve VAW and survivors have few alternatives but to interact with police when in need (Conroy et al., 2021; Department of Justice Canada, 2021; Leisenring, 2012). There is little empirical research that has explicitly consulted survivors on this matter.

This research contributes to understanding survivors’ concerns with police use of BWCs by highlighting the voices of women but it is important to acknowledge the limitations of the research. First, the data presented here is produced from a non-probability sample. As such, the findings best represent the attitudes of the persons who participated in the research and should not be assumed to generalize beyond this group. Second, the sample included persons who did not interact with officers wearing BWCs. As such, while all participants identified as survivors of VAW and have valuable insight on how BWCs may affect them as a survivor, this characteristic of the sample should not be conflated with the characteristic of actually having interacted with an officer wearing a BWC. Finally, considerably more cases would be needed to begin to explore patterns associated with intersectional identity characteristics of importance such as race/ethnicity. Our work does not comment on such details because we did not feel we had a sufficient sample to warrant doing so. It is important to recognize that perceptions of BWCs may vary according to women’s intersecting social locations. These intersecting social locations and their accompanying structural inequalities shape encounters with and perceptions of the police (Cochran & Warren, 2012). For example, many immigrant and racialized women do not call police because they fear police will be racist or culturally insensitive (Couture-Carron et al., 2021). It is possible that racialized women may see BWCs as a mechanism for improving their treatment and increasing their (and their abuser’s) safety when police contact becomes necessary. Alternatively, women from racialized communities are already overpoliced and over-surveilled (Hattery & Smith, 2021; Sewell et al., 2016). Being filmed while reporting may exacerbate feelings of police scrutiny. Given that most participants in this study were White, racialized women’s experiences may not be represented in this study. Future research in this area should focus on the “most marginalized” survivors (see Durfee, 2021) to understand this vulnerable group’s perceptions of the potential benefits and dangers of police use of BWCs.

This study offers insight into concerns that survivors have with BWCs. Specifically, survivors are concerned with how trauma may appear on BWC footage and how that could be used against them, the potential for BWCs to undermine survivors’ comfort and reporting, and that BWCs may contribute to survivors’ revictimization and loss of control. Given that women already hesitate to report their victimization to police, knowledge of these concerns is imperative to continuing to improve survivor–police relations. Since many women only reach out to police when they are in a life-threatening situation (Bonomi et al., 2006; Johnson, 2015), practices that may discourage reporting may have life and death consequences for women. Practices that have the potential to contribute to survivors’ revictimization are also concerning given the implications for survivors’ well-being and willingness to seek further help (Campbell & Raja, 2005). In short, survivor–police relations are often tenuous. This fragility necessitates understanding the potential impact of policing innovations, including BWCs, on these relations. BWCs are a technology that the survivors in this study conveyed support for and see promise in, but BWCs also present concerns for survivors that warrant attention.

## Notes

1. As of 2019, 17 Canadian police services had trialed BWCs but most pilots had been discontinued, while by 2022, 36 services were actively using BWCs (Saulnier & Abbatangelo, 2024).

2. See Saulnier et al. (2022) for a detailed discussion of why survivors support police use of BWCs and recommendations on the rules that should accompany police use of BWCs to support victim sensitive practices.

3. The report narrowly focuses on survivors abused by a current or former legally married or common-law partner.

4. This study was approved by Lakehead University’s Research Ethics Board [#122629] on March 20, 2019.

5. All names are pseudonyms.

6. Officers are typically not required to receive consent to activate a BWC with some exceptions (e.g., when in a dwelling house without exigent circumstances, in a hospital).
